# Supplementary material for: Gene prioritization based on random walks with restarts and absorbing states, to define gene sets regulating drug pharmacodynamics from single-cell analyses
Source: PLoS One. 2022 Nov 7;17(11):e0268956. doi: 10.1371/journal.pone.0268956 (PMC9639845; doi:10.1371/journal.pone.0268956)
Supplement: S1 File — (PDF) [file pone.0268956.s001.pdf]

6 Supporting information: results

6.1 Individual scores and their symmetry

Global analysis. We first notice that the scores $Q^{(r)}(x, p)$ and $Q^{(r)}(p, x)$ for all pairs $X \times P$ tend to be sharply peaked near the origin, especially for small values of the restart rate r (Section 6.1, Fig. S1).

To study the symmetry of scores, we resort to scatter plots whose x and y axis are the ranks of scores (Eq. 4), and values displayed are the scores $\log(Q^{(r)}(x, p))$, $\log(Q^{(r)}(x, p))$ and their difference $\log(\mid Q^{(r)}(x, p) - Q^{(r)}(p, x) \mid)$ (Fig. S2).

Upon inspecting these plots by varying r, the following appears:

- (Scatter plots, scores for $X \rightsquigarrow P$ i.e. first column) Plotting the value for ST results in a narrow vertical band of large values – consistent with the fact that the histogram of log values is sharply peaked near zero yielding large negative logs.
- (Scatter plots, scores for in $P \rightsquigarrow X$ i.e. second column) Likewise, plotting the value for $X \rightsquigarrow P$ results in a narrow horizontal band, but we notice a higher density for large ranks in TS.
- (Comparing rows) Increasing the restart rate widens the range of scores (Fig. S1), which in turn stresses the aforementioned vertical and horizontal bands.
- The previous observations are combined on the difference plot (Fig. S2 (Right column)), and are especially salient for $r = 0.3$. It indeed appears that large values of the log of the difference are only obtained for a small rank in $X \rightsquigarrow P$ (large score in $X \rightsquigarrow P$) or in $P \rightsquigarrow X$ (large score in $P \rightsquigarrow X$); moreover, large negative values of the log are not observed near the origin, which shows that large scores in $X \rightsquigarrow P$ and $P \rightsquigarrow X$ are not observed concomitantly except for ranks ≤ 50.

This lack of symmetry of scores is a strong indication that paths joining x to p have significantly different features from those joining p to x, in particular in terms of high degree vertices. Every path from x to p is also a path from p to x. However, a high degree vertex which appears early in the path from x to p appears late in the reverse path. Such high degree vertices yield many alternative paths, which are more competitive with each other in the x to p direction.

Incidence of the size of X on scores and their symmetry. We study the incidence of the sizes $|X|, |P|$ on the symmetry score ratio $H(I)$ of Eq. (7). To do so, we pick random subsets $X' \subset X$ of size $\{|P|, 110, 220\}$, performing 1000 repeats for each value (Fig. S3).

First, considering the statistics for the symmetry ratio $H(I)$, despite seemingly related values for the mean and std deviation of the statistic $H(I)$ for the two restart rates $r = 0.01$ and $r = 0.3$, the p-value of the non-parametric two-sample test used shows a strong evidence to reject the equality of distributions. Second, $H(I)$ displays a marked dependence on the size of X, which is related to two facts. On the one hand, increasing the size of X does not affect the score in the direction $X \rightsquigarrow P$; however, the hitting probabilities in the direction PX are getting *diluted*. On the other hand, due to the small-world nature of the graph used,

increasing the size of X could result in high degree nodes close to vertices in P, with the consequence discussed above.

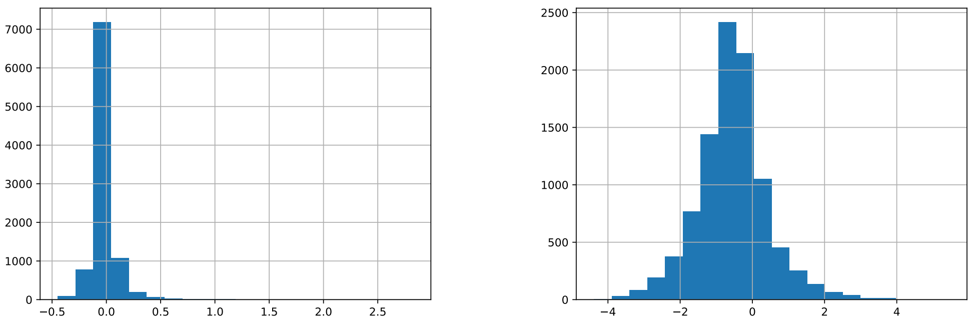

$r = 0.01$ $r = 0.3$

Figure S1: **MINT: histogram of log scores for direction $X \rightsquigarrow P$.**

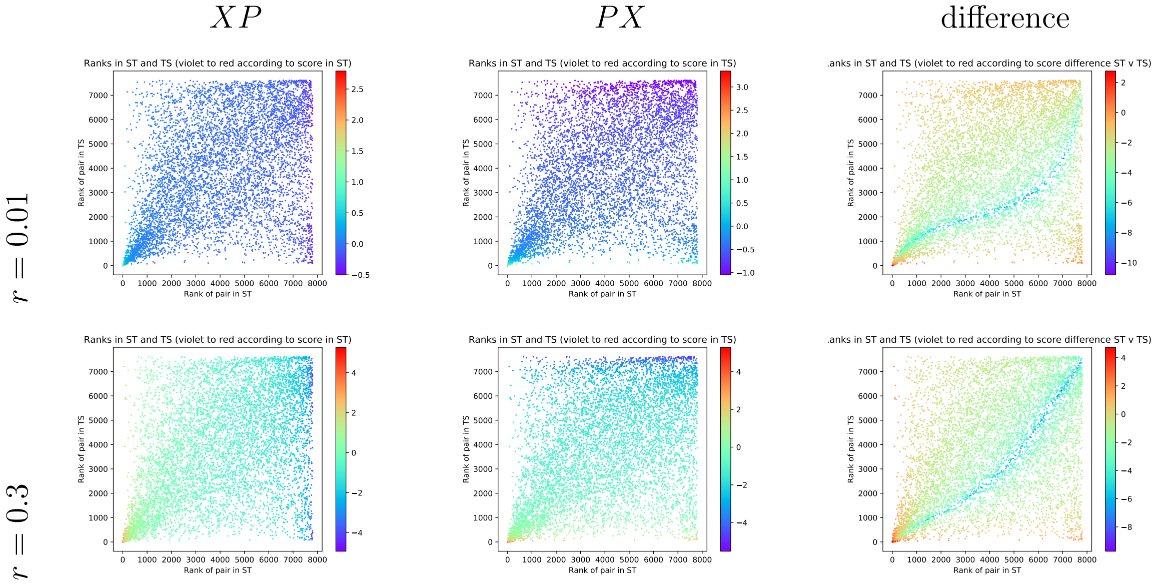

Figure S2: **MINT: Scatter plot of log scores for the directions $X \rightsquigarrow P$ and $P \rightsquigarrow X$** Color coding of point is as follows: **(Left)** $\log Q^{(r)}(x,p)$ **(Middle)** $\log Q^{(r)}(x,p)$ **(Right)** $\log \mid Q^{(r)}(x,p) - Q^{(r)}(p,x) \mid$

6.2 Saturation indices and hits

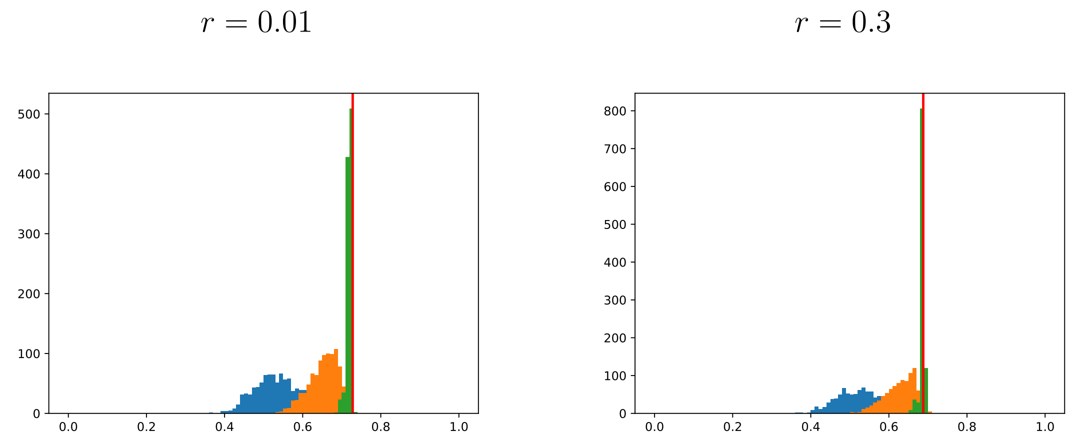

	$\|X'\| = \|P\|$	$\|X'\| = 110$	$\|X'\| = 220$	224
	(μ, σ)	(μ, σ)	(μ, σ)	H(I)
$r = 0.01$	$(0.54, 0.063)$	$(0.65, 0.037)$	$(0.72, 0.005)$	0.728072
$r = 0.3$	$(0.53, 0.064)$	$(0.63, 0.039)$	$(0.69, 0.006)$	0.688025
p-values	8.14e-3	6.72e-38	∼0.0	NA

Figure S3: **Symmetry score ratio $H(I)$ (Eq. 7) at the instance level.** Subsets $X' \subset X$ of size $s \in \{|P|, 110, 220\}$ are used, $N_r = 1000$ repeats for each size. **(Figures)** Distributions of the statistic $H(I)$ for two restart rates $r = 0.01$ and $r = 0.3$: blue ($|X'| = |P|$) orange ($|X'| = 110$) green ($|X'| = 220$). Red lines represent the values of $H(X)$. **(Table)** Statistical summaries μ and σ for $H(I)$. The p-value reported is that of the Mann-Whitney U test, two-sided alternative. The last column of the table corresponds to the complete gene set X.

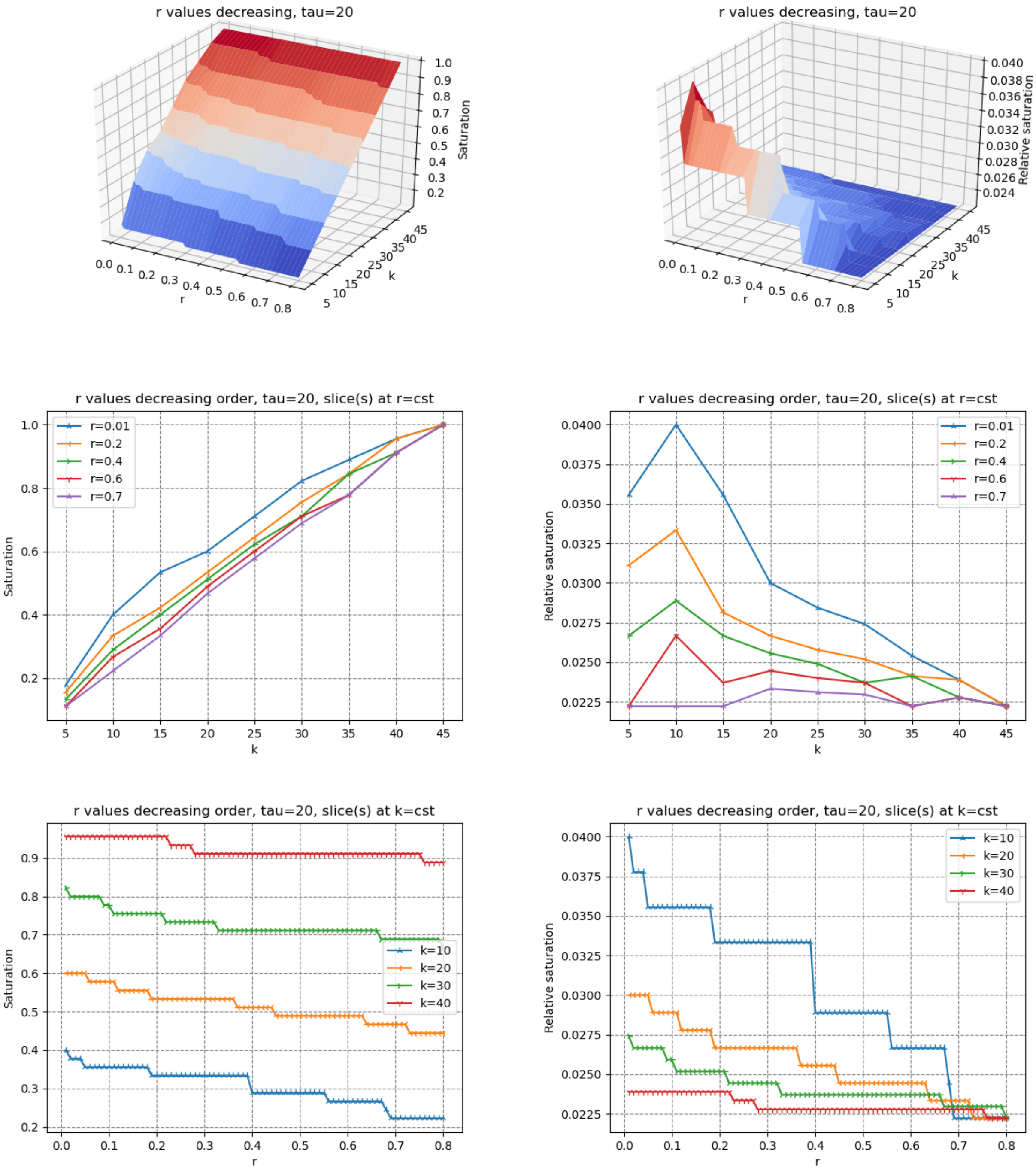

Figure S 4: **(Genetrank-renorm) Saturation plots (Def. 5) for $\tau = 20$. Values of r processed in decreasing order. (Left column)** Saturation index and slices at $r = cst$ and $k = cst$ (See Eq. 9) **(Right column)** Relative saturation index and slices at $r = cst$ and $k = cst$ (See Eq. 10)

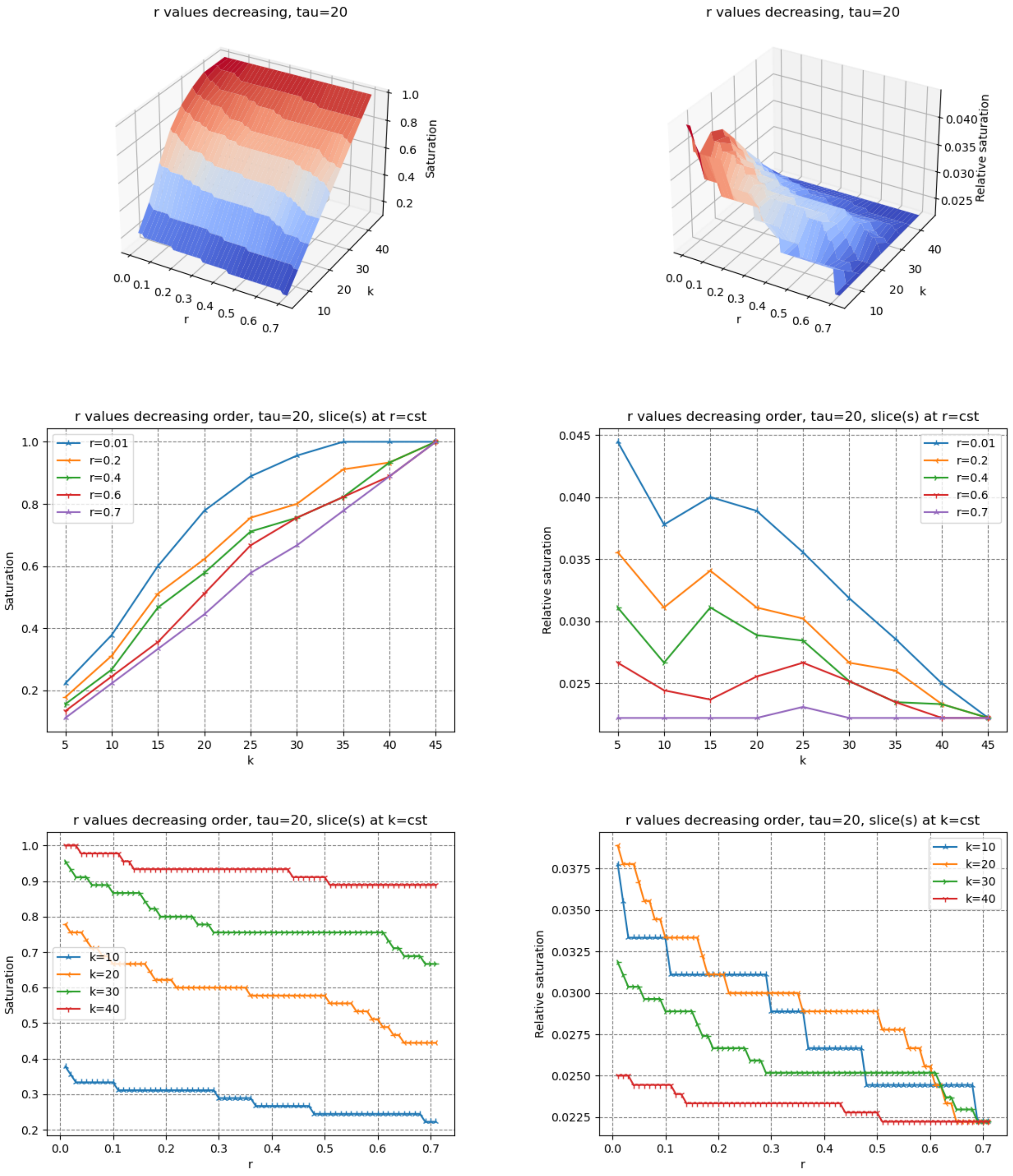

Figure S5: **(Genetrank-AS) Saturation plots (Def. 5) for $\tau = 20$. Values of r processed in decreasing order. (Left column)** Saturation index and slices at $r = cst$ and $k = cst$ (See Eq. 9) **(Right column)** Relative saturation index and slices at $r = cst$ and $k = cst$ (See Eq. 10)

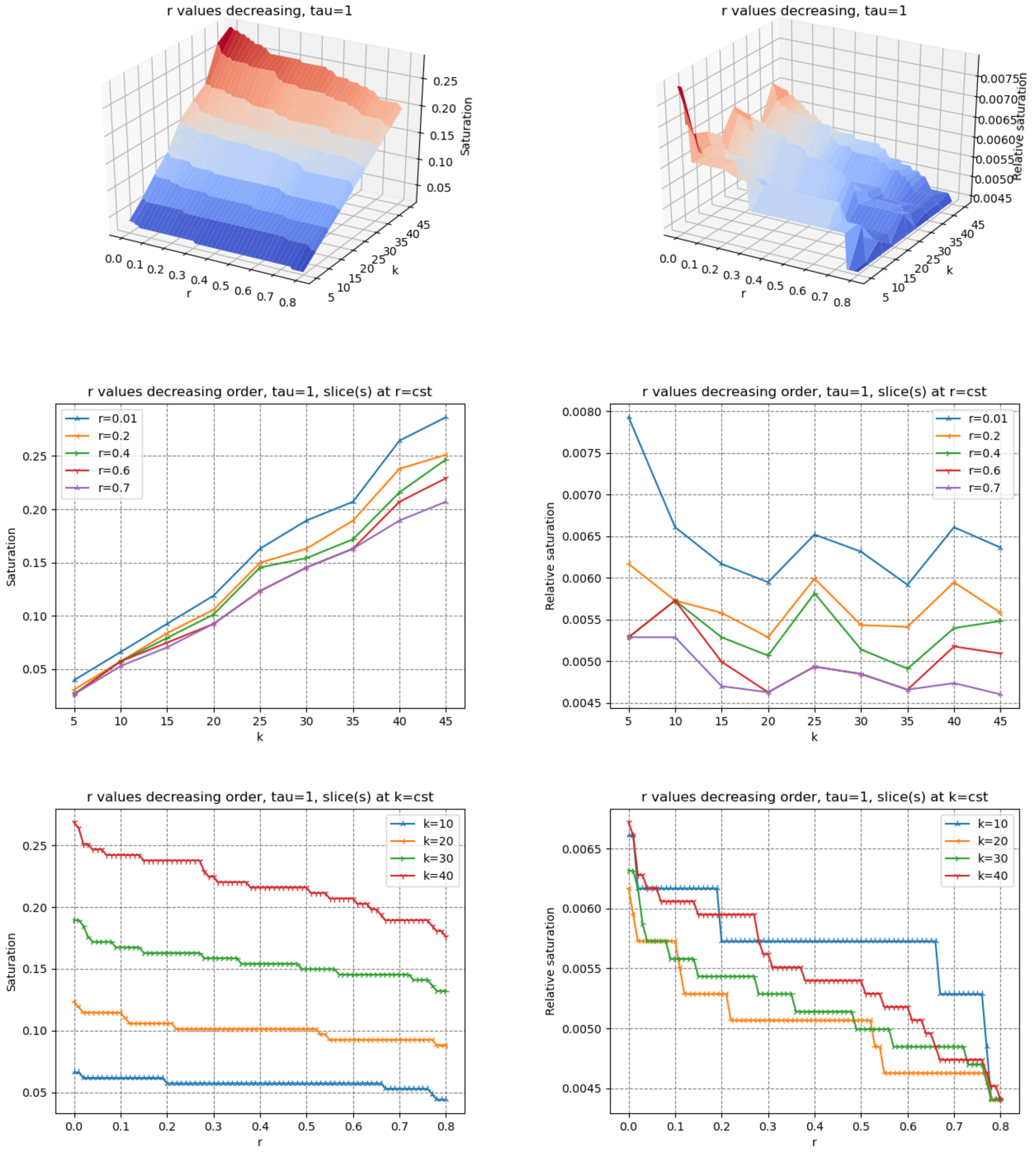

Figure S 6: **(Genetrank) Saturation plots (Def. 5) for $\tau = 1$. Values of r processed in decreasing order. (Left column)** Saturation index and slices at $r = cst$ and $k = cst$ (See Eq. 9) **(Right column)** Relative saturation index and slices at $r = cst$ and $k = cst$ (See Eq. 10)

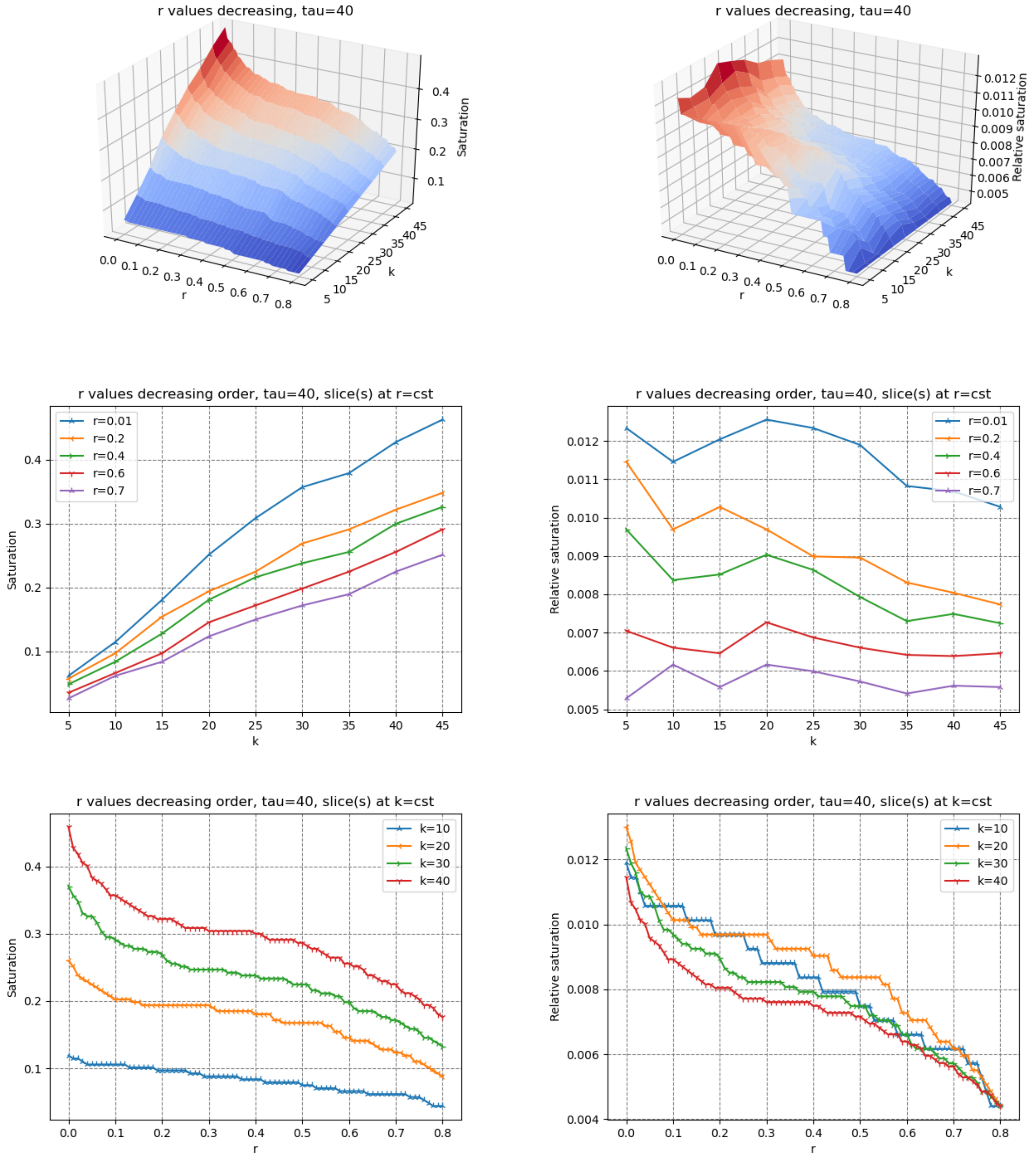

Figure S7: **(Genetrank) Saturation plots (Def. 5) for $\tau = 40$. Values of r processed in decreasing order. (Left column)** Saturation index and slices at $r = cst$ and $k = cst$ (See Eq. 9) **(Right column)** Relative saturation index and slices at $r = cst$ and $k = cst$ (See Eq. 10)

6.3 Hits

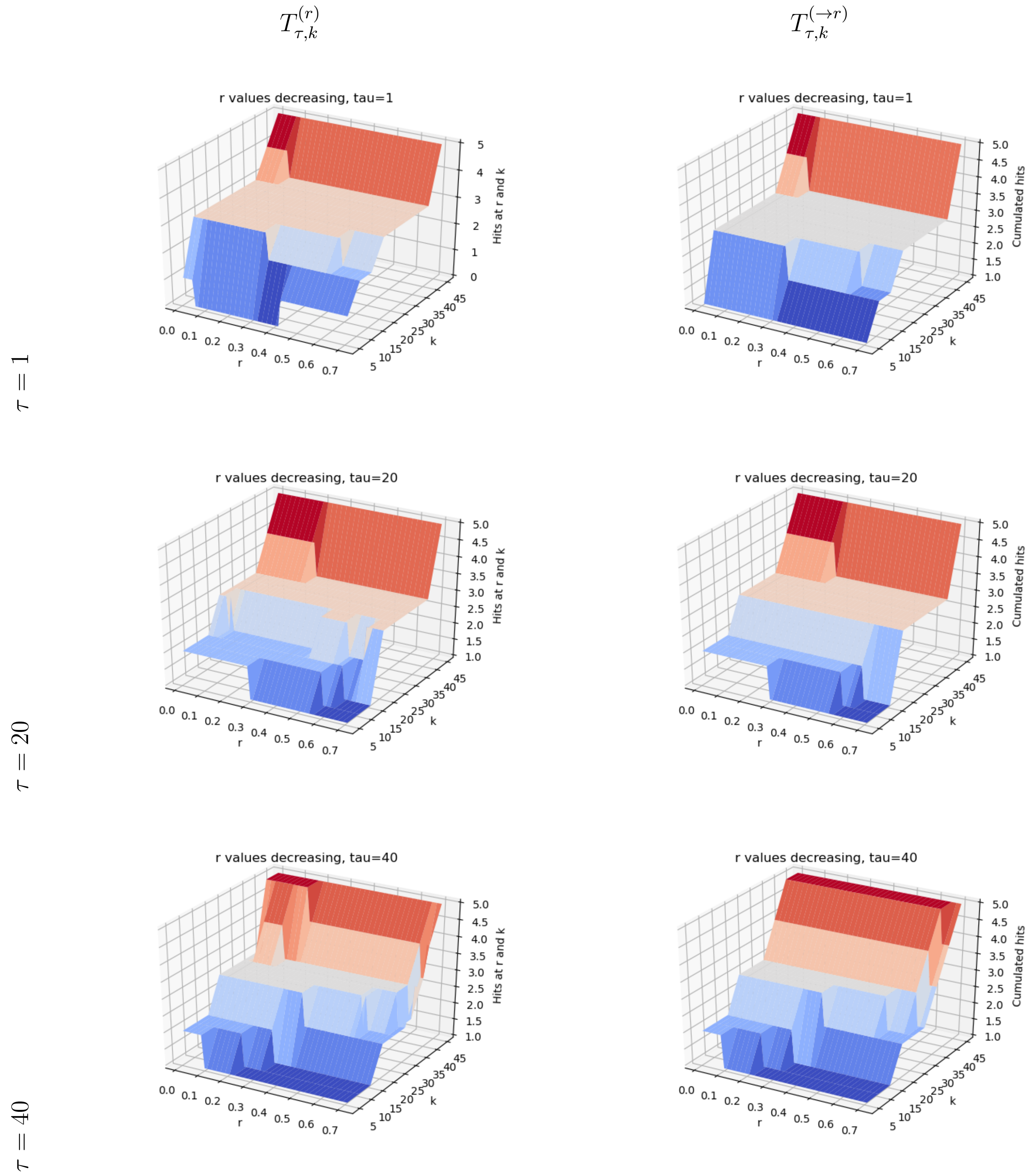

Figure S8: **(Genetrank-renorm) Hits (Def. 6) for the list of reference genes O15304 (SIVA1), P78537 (BLOC1S1), P0CW18 (PRSS56), P53007 (SLC25A1), Q9Y2X8 (UBE2D4), DNM1L(O00429).**

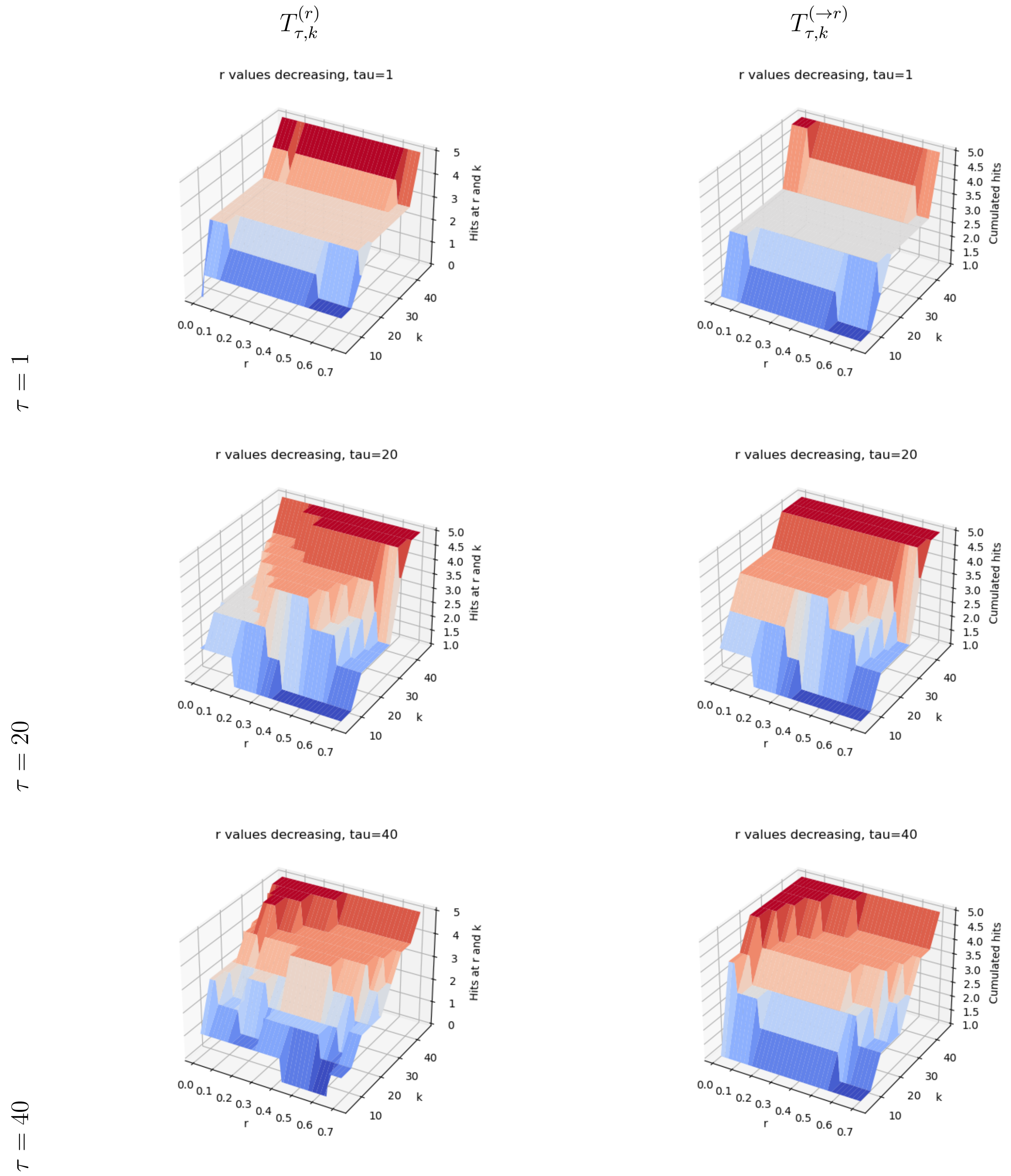

Figure S9: **(Genetrank-AS) Hits (Def. 6) for the list of reference genes O15304 (SIVA1), P78537 (BLOC1S1), P0CW18 (PRSS56), P53007 (SLC25A1), Q9Y2X8 (UBE2D4), DNM1L(O00429).**

6.4 Deferentially expressed genes

GeneID	ProtID
SLC25A1	P53007
PTMS	P20962
CHD7	Q9P2D1
ACTN4	O43707
MAP2K1	Q02750
UBE2D4	Q9Y2X8
MUL1	Q969V5
ICMT	O60725
DNM1L	O00429
BLOC1S1	P78537
LGALS3BP	Q08380
SEC63	Q9UGP8
ALDH1B1	P30837
POR	P16435
CMAS	Q8NFW8
NPC2	P61916
JADE1	Q6IE81
ANAPC5	Q9UJX4

Table S 1: **Set of 18 genes obtained by intersecting the list of 65 genes yielded by edgeR, with this list yielded by `Genetrank` ($k = 50$, range of values of $r : 0..0.8$, $\tau = 41$).**
